# Functional Diversity of Neuronal Cell Adhesion and Recognition Molecule L1CAM through Proteolytic Cleavage

**DOI:** 10.3390/cells11193085

**Published:** 2022-09-30

**Authors:** Irina I. Stoyanova, David Lutz

**Affiliations:** 1 Department of Anatomy and Cell Biology, Faculty of Medicine, Medical University, 9002 Varna, Bulgaria; 2 Department of Brain Ischemia Mechanisms, Research Institute, Medical University, 9002 Varna, Bulgaria; 3 Department of Neuroanatomy and Molecular Brain Research, Ruhr University Bochum, 44801 Bochum, Germany

**Keywords:** cell adhesion and recognition, proteolysis, L1, NCAM, ectodomain shedding

## Abstract

The neuronal cell adhesion and recognition molecule L1 does not only ‘keep cells together’ by way of homophilic and heterophilic interactions, but can also promote cell motility when cleaved into fragments by several proteases. It has largely been thought that such fragments are signs of degradation. Now, it is clear that proteolysis contributes to the pronounced functional diversity of L1, which we have reviewed in this work. L1 fragments generated at the plasma membrane are released into the extracellular space, whereas other membrane-bound fragments are internalised and enter the nucleus, thus conveying extracellular signals to the cell interior. Post-translational modifications on L1 determine the sequence of cleavage by proteases and the subcellular localisation of the generated fragments. Inside the neuronal cells, L1 fragments interact with various binding partners to facilitate morphogenic events, as well as regenerative processes. The stimulation of L1 proteolysis via injection of L1 peptides or proteases active on L1 or L1 mimetics is a promising tool for therapy of injured nervous systems. The collective findings gathered over the years not only shed light on the great functional diversity of L1 and its fragments, but also provide novel mechanistic insights into the adhesion molecule proteolysis that is active in the developing and diseased nervous system.

## 1. Introduction

Neural cell adhesion molecules, also called cell recognition molecules, belong to an integral membrane protein superfamily with characteristic adhesive and signalling properties [1]. In particular, two well-studied members of the family, the cell adhesion molecule L1CAM (or simply L1) and the neural cell adhesion molecule NCAM, are crucial for cell migration, proliferation, and differentiation during the early stages of nervous system formation, as well as postnatally in adult neurogenesis [2,3,4] and neural plasticity [5,6]. Data generated over the past twenty years suggest that, at the protein level, cell adhesion molecules can exist in the form of proteolytic fragments. Here, we focus on the intracellular distribution of membrane-bound fragments of L1 formed after the application of particular stimuli. We present contemporary approaches to understanding the functions of these fragments, together with the proteases that generate them, in the context of nervous system development and pathology.

## 2. L1CAM in Nervous System Development and Neuropsychiatric Disease

L1 has been described as a cell recognition molecule [7], which facilitates adhesion between neurons. During development, L1 is required for the outgrowth, fasciculation, and guidance of axons, glial process formation, and neuronal migration [8,9,10]. At the adult stages, L1 is involved in neural plasticity, the consolidation of learning and memory, and post-injury regeneration [11,12,13,14]. The L1 gene of the X–chromosome in humans encodes a transmembrane type I protein comprising 1257 amino acids. The importance of L1CAM for the proper development of the nervous system is highlighted by a plethora of reported pathological mutations in this gene [15]. Many of these mutations cause severe neurological deficits, often leading to the premature deaths of the affected individuals [15]. Four human X-linked neurodevelopmental pathologies comprise the L1 syndrome: hydrocephalus, the degeneration or lack of the corpus callosum, spastic paraplegia, and intellectual disability [16,17,18]. Mice deficient in L1 display similar anatomical abnormalities, resulting in severe behavioral deficits [19,20,21,22,23]. In these mice, a considerable number of corticospinal tract axons do not cross the midline to the opposite dorsal column [24]. The aberrantly misplaced ipsilateral axons do not project beyond cervical levels [24]. Hence, abnormal pyramidal decussation and ataxia are often seen in L1-deficient mice [9]. Similar neuroanatomical alterations have been found in mice carrying the constitutive loss-of-function mutation p.C264Y in the murine L1CAM gene. The mutation is also pathogenic in L1 syndrome patients [25]. Importantly, ectopic expression of L1 in astrocytes has been shown to affect corticospinal tract development [26]. It has been suggested that defective axonal projections through the corpus callosum or corticospinal tract may result in neuronal cell death, loss of cortical gray matter, increased brain compliance, and, thus, enlarged ventricles [27]. Strikingly, domain modelling has predicted that some missense mutations in L1CAM lead to protein misfolding and accumulation in the endoplasmic reticulum with aberrant cell surface expression [16,17,28,29,30]; meanwhile, other types of mutations affect specific amino acids in the L1CAM protein, which undergo molecular interactions [31] (Bateman et al. 1996). Not all of the molecular and cellular mechanisms underlying pathogenic mutations have been explored, but they surely manifest as part of L1 syndrome.

## 3. The Structure and Functions of Cell Adhesion Molecule L1

**Figure 1:**
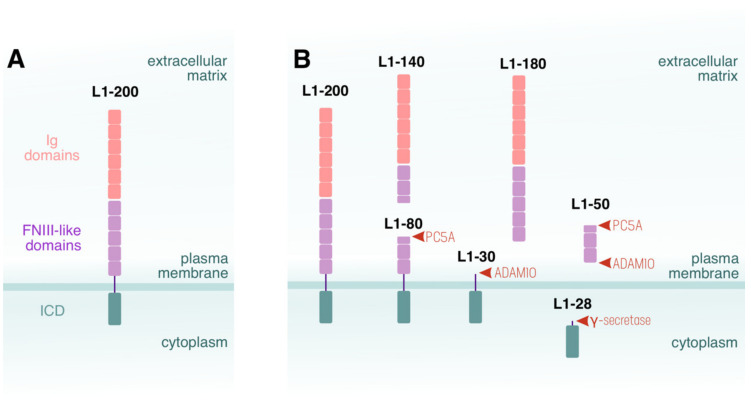
Structure and ectodomain shedding of L1. (**A**) Full-length L1 (L1-200) consists of six Ig-like domains, five FNIII-like repeats, a transmembrane, and an intracellular domain. (**B**) PC5A cleaves L1-200 within the third FN repeat to generate a membrane-bound 80 kDa (L1-80) and a soluble 140 kDa (L1-140) fragment. L1-200 and L1-80 are substrates of the ADAM10 protease, which generates a membrane-bound 32 kDa (L1-32) and a soluble 180 kDa (L1-180) or a soluble 50 kDa (L1-50) fragment. L1-32 can be processed by the γ-secretase to an intracellular 28 kDa (L1-28) fragment.

The protein backbone of L1 consists of a short and highly conserved cytoplasmic domain, a transmembrane part, and an extracellular region formed by six immunoglobulin (Ig)-like and five fibronectin-type III (FNIII)-like domains [32], as shown in Figure 1A. L1 binds homophilically to other L1 molecules [33] or heterophilically to distinct binding partners [34,35,36]. At the plasma membrane of a cell, the ectodomain of L1 can interact with other proteins in a ‘cis’ configuration [37], while the ‘trans’ interaction mediates cell–cell contacts [38]. It seems that the ectodomain of L1 is a lectin that interacts with sialic acid [39]. There are several potential sites on L1 for glycosylation, which can affect homo- and heterophilic interactions [40,41]. Furthermore, L1 can undergo ubiquitination at its C-terminus [42]; however, these post-translational modifications of the adhesion molecule, and their functional significance for the proper formation of the nervous system, are not well understood.

Initially, L1 was found in the nervous system, and has been thoroughly studied. However, the molecule has been also detected in different types of cancer [43]. In patients with solid carcinoma, L1 is overexpressed [43], and high expression of L1 in these tumours predicts a poor outcome [44,45,46]. In tumours, L1 rarely exerts ‘adhesive properties’ in terms of holding tumour cells together; rather, it induces invasive and aggressive tumour growth, metastasis, and chemoresistance [36,47,48,49]. L1 shows abnormal expression in the blood vessels of a variety of malignant tumours and has been related to a multitude of pro-angiogenic effects [50]. Notably, Angiolini and colleagues discovered a novel isoform of L1CAM expressed in endothelial cells as a result of a NOVA2-induced (neuro-oncological ventral antigen 2) alternative splicing removing the exon coding for the transmembrane domain of L1 [51]. This isoform represents a soluble L1CAM variant, which is released by endothelial cells and able to stimulate angiogenesis via autocrine/paracrine mechanisms [51]. This isoform is overexpressed in the vasculature of ovarian cancer, and high expression levels correlate with ovarian cancer aggressiveness [51]. The L1 isoforms created by alternative splicing are just a small portion of those that contribute to the diversity of L1 entities circulating in the body. At the protein level, for instance, L1 is cleaved within the ectodomain, and in carcinoma patients this cleavage results in the shedding and accumulation of soluble forms into the extracellular space, including serum and ascites [43,47,52,53], as shown in Figure 1B. Another proposed mechanism for the metastasising of cancer cells is the recruitment of L1 and the activation of the mechanotransduction effectors, such as Yes-associated protein (YAP) and myocardin-related transcription factor (MRTF). YAP activation is mediated via β1 integrin and integrin-linked kinase (ILK), which facilitate the formation of metastasis-triggering cells [54]. However, it is still unclear why and how L1 is involved in the development of aggressive tumours on the one hand, and in the normal functioning of the nervous systems on the other. These lines of thought are mirrored by studies reporting increased L1 fragments in the cerebrospinal fluid of patients with Alzheimer’s disease [55].

Several proteases are involved in the process of the ectodomain shedding of L1 (Figure 1B); these are mainly members of the ADAM (a disintegrin and metalloproteinase) family, and include ADAM10, ADAM7, and BACE1 [53,56,57,58] (for a more detailed review, see Linneberg et al. [59]). However, serine proteases, such as plasmin [60] can also participate. Plasmin and trypsin, as well as the pro-protein convertase 5A (PC5A), cleave L1 within the third FNIII-like repeat [61] to generate transmembrane, intracellular, and soluble extracellular fragments. Members of the ADAM family also contribute to the formation of those fragments [53,62,63], releasing the entire ectodomain of L1 [56] (Figure 1B). This phenomenon has been observed not only during brain development but also in tumour cells in vitro [64]. Presenilin and beta-secretase generate an intracellular L1 fragment, found in the nucleus, where it probably influences gene expression [63] (Figure 1B). The proteolysis of L1 contributes to post-translational diversity, which obviously dominates over the genomic diversity reported for L1CAM so far.

L1 carries the carbohydrate Lewis^X^ [65], which is crucial for the development and further functioning of the nervous system, and particularly for neurite outgrowth and myelination [66]. Lewis^X^ is involved in the processing of L1 by proteases, and myelin basic protein (MBP) was identified as a serine protease for L1, which interacts with L1 in a Lewis^X^-dependent manner [67,68]. MBP is a major myelin constituent and has a clinical implication in demyelinating diseases, such as multiple sclerosis [69,70,71,72,73]. *Shiverer* mice, which are deficient in MBP, show a progressive disorder characterised by tremors and seizures, leading to early death [69,74]. Surprisingly, neurons show that MBP reactivity is similar to that of myelin-producing cells [75]. After the L1-specific immunostimulation of cultured murine cerebellar neurons, MBP is released into the culture medium as a sumoylated dynamin-containing protein to cleave L1 at R687 (targeted also by trypsin and plasmin) in the extracellular domain, thus yielding a transmembrane 70 kDa L1 fragment (L1-70) [61,68]. MBP plays a major role in the generation of this fragment, since it is abolished when MBP is manipulated in a variety of ways, such as by genetic ablation (in *shiverer* mice) or the mutagenesis of the proteolytically active or cleavage sites, as well as by the application of serine protease inhibitors. The MBP-mediated generation of L1-70 promotes neurite outgrowth and the survival of neurons, as shown in vitro. Interestingly, in dissociated cerebellar neurons from wild-type and MBP-deficient *shiverer* mice, and when cultured in a medium supplemented with the MBP antibody or L1 holding the MBP cleavage site, the formation of neurites and neuronal survival is hampered [67,68]. The MBP-dependent L1-70 also promotes Schwann cell proliferation and myelination in cultured dorsal root ganglion neurons. These combined findings provide evidence for novel functions of the Lewis^X^-based interaction between L1 and MBP in the nervous system [67,68].

Further studies on the relevance of the proteolytic activity of MBP on L1 in vivo, in the developing spinal cord, have identified the proteolytically active site in MBP [67]. A serine residue of MBP mutated by a genetic nucleotide exchange disrupted MBP’s proteolytic activity and abolished the L1-dependent cellular responses when applied to cultured neurons. The administration of adeno-associated viral particles that encode proteolytically active MBP into *shiverer* embryos in utero prevented the manifestation of all the developmental spinal cord abnormalities mentioned above. However, these parameters become abnormal after the in utero injection of proteolytically inactive MBP. These findings suggest that the serine protease MBP acts on L1 to facilitate important morphogenic events during the early stages of nervous system development [67].

In addition to its essential role in the formation of the nervous system, L1 also stimulates recovery processes in animal models of acute and chronic neurodegenerative entities [76]. Does the proteolysis of L1 affect the regeneration of the nervous system after acute trauma? Using the spinal cord and femoral nerve injury paradigms of adult mice, it has been investigated whether MBP, which is proteolytically active on L1 in the third FNIII domain, would affect regeneration [67,68]. The treatment of the injured spinal cords and femoral nerves of non-mutant mice with active recombinant MBP leads to the elevation of L1 levels, the restoration of the structural integrity, and the improvement of functional performances. It is noteworthy that the immunosuppression of MBP with a specific antibody at the site of the injury leads to impaired regeneration. These opposing effects have also been achieved by injecting viruses that encoding either proteolytically active or inactive MBP at the injury area. The results from these experiments reveal that MBP has another L1-mediated ability, which could be used for the treatment of acute injuries of the nervous system.

In the search for other early proteases/binding partners that target L1, the extracellular matrix protein Reelin has been identified as interacting with cell adhesion molecule L1 [77]. Reelin seems to stimulate the underlying neuronal relocation of signalling pathways by interacting with lipoprotein receptors [78,79,80,81,82,83], probably acting as a protease [84,85,86]. Interestingly, Reelin itself is a substrate for metalloproteases [87,88,89,90,91], which cleave the protein into different fragments [92,93,94]. Data continue to accumulate regarding the Reelin fragments and their functions, but they remain enigmatic [95,96,97,98]. Thus, it has been found that, in addition to the full-length Reelin, the N-R2 and N-R6 terminal fragments also bind to L1. However only full-length Reelin and the N-R6 fragment mediate the cleavage of L1 (within the diabasic sequence _858_RKHSKR_863_) and the appearance of an 80 kDa fragment (L1-80), and stimulate the migration and axonal outgrowth of dissociated cortical and cerebellar neurons [77]. Remarkably, in the early stages of brain cortex development, the expression of the N-R6 fragment parallels the generation of L1-80. Furthermore, because newly generated neurons migrate toward the Reelin-containing marginal zone, Reelin has been considered to be a guiding signal [99]. On the other hand, Reelin might be a stop signal [99], since the migrating neurons in Reelin-deficient (*reeler*) mice invade the marginal zone, unlike the cells of the wild-type mice. It is therefore worth mentioning that the developing L1-deficient cerebral cortex displays morphological abnormalities in layer formation, partially overlapping with those seen in the cerebral cortices of the *reeler* mice. However, in utero electroporetic administration of L1-80 into the cortices of *reeler* embryos normalises neuronal migration [77]. These findings point to the significance of the interaction between L1, Reelin, and the Reelin-mediated formation of L1-80 during the early stages of brain development. Unlike L1-80, the full-length L1 fails to induce neuronal migration in Reelin-deficient mutants [77]. Thus, the combined findings reveal that, as soon as L1 is proteolytically cleaved, cell motility occurs. Moreover, studying L1 fragments provides deeper insights into the function of the proteases that process L1. These findings provide evidence that the cleavage of L1 contributes to different L1 functions. How and when the L1 fragments perform all these functions is still not well understood.

In this respect, it is important to mention that another member of the CAM family, the neural cell adhesion molecule NCAM, is prone to proteolysis in a similar fashion to L1. NCAM is crucial not only for the proper development of the nervous system, but also for maintaining the high cognitive functions of the adult brain (for further details, see [3]). Similarly to L1, NCAM is proteolytically cleaved by several proteases into extracellular, transmembrane, and intracellular fragments [100,101]. NCAM is post-translationally modified to carry the glycan polysialic acid (PSA), which strongly influences the functions of NCAM. PSA–NCAM is upregulated in tumour cells [102] and has been considered to be an adverse prognosis factor in glioblastoma [103]. There are fluctuations in the levels of PSA–NCAM in the suprachiasmatic nucleus [104], and genetic deletions of NCAM and PSA have been shown to impair circadian functions [105]. Recently, two new PSA-binding proteins, positive factor 4 (PF4) and cofilin, have been recognised as being responsible for the nuclear import of PSA-carrying NCAM fragments. PF4 and cofilin are involved in RNA polymerase II-dependent transcription and, as such, they can modulate gene expression: the PSA-carrying NCAM fragment increases mRNA and protein expression of the nuclear receptor subfamily 2 group F member 6, whereas the PSA-lacking NCAM fragment increases low density lipoprotein-receptor-related protein 2 and α-synuclein [106]. These combined data produce a two-sided story, revealing that CAM cleavage and post-translational modifications play an important role in the proper development of the nervous system, but that, when out of control, they are also a hallmark of pathological change. The types of proteases involved hereby determine what kind of molecular fragments will be generated, and what their destiny will be, thus modulating a plethora of intra- and extracellular events. Therefore, we can speculate that proteolysis is a key mechanism in the production of significant functional diversity amongst the members of the adhesion molecule family.

## 4. Stimulation of Proteolysis and the Intracellular Trafficking of Proteolytic Fragments

**Figure 2:**
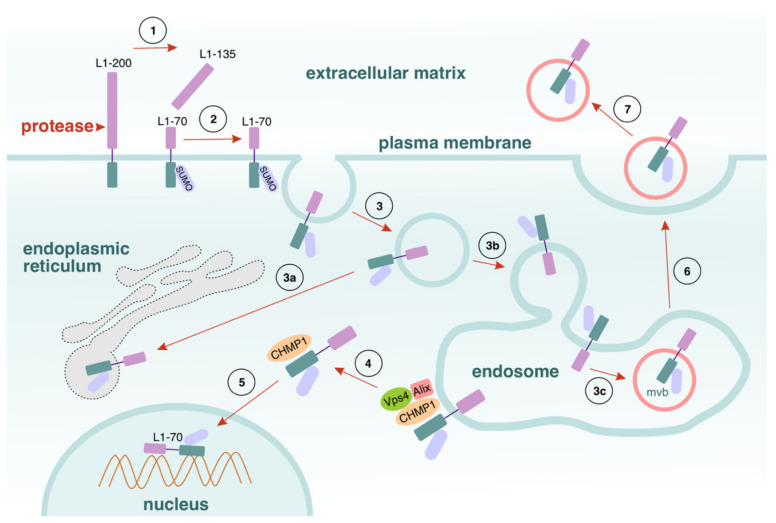
The formation, internalization, and intracellular pathways of L1-70. (1) Full-length L1 (L1-200) is cleaved by (serine) proteases into a membrane-bound 70 kDa L1 fragment (L1-70) and a soluble 135 kDa fragment (L1–135). (2,3) Via endocytosis, L1-70 enters the cytosol and it is then transported either to the endosomes or to the endoplasmic reticulum (3a, 3b). L1-70 is distributed to the endoplasmic reticulum, the sorting endosomes, and the late endosomes. (3c) L1-70 is loaded onto multivesicular bodies (mvb). (4) Once released from the endosomes, a process that depends on the ESCRTIII proteins Alix, Vps4, and CHMP1, and on a conjugation with CHMP1, L1-70 is translocated into the nucleus and associates with the chromatin (5). (6) Another possible direction for L1-70 trafficking is via exocytosis (7).

The homophilic and heterophilic interactions of L1 can stimulate signal transduction pathways, generating cellular responses. As shown previously [107], the stimulation of signalling by function-triggering L1 antibodies or by the ectodomain of L1 fused with the Fc part of human IgG_1_ activates the cleavage of L1 by a serine protease at the plasmalemma. This yields a sumoylated transmembrane L1 part of approximately 70 kDa (see [107] and [61]), which harbours the intracellular and transmembrane domains, as well as part of the extracellular domain (Figure 2).

After generation, this transmembrane fragment is internalised to a late endosomal area, then further shifted consecutively to the cytoplasm and the nucleus. Having been released from the endosomal membranes into the cytoplasm, the fragment is then further transferred to the nucleus under the control of importin and chromatin-modifying protein 1. There are two motifs in L1 that are crucial for this process: a sumoylation site at K_1172_ and a nuclear localisation signal related to K_1147_. When both are mutated, the L1-stimulated generation and nuclear import of the 70 kDa fragment is abolished. It has been found that the nuclear 70 kDa L1 fragment is associated with the chromatin-rich nuclear fraction of neurons, implying that the nuclear import of the fragment, and hence, the possible association of the fragment with DNA, may affect gene expression. Furthermore, the expression of this 70 kDa L1 fragment varies over the course of the formation of the nervous system, as well as when acute and chronic injuries are sustained in adulthood [55]; the fragment has been suggested to be a key player in those processes [108,109]. Moreover, it can be speculated that the fragment might also take part in tumorigenesis, because proteases are also adversely upregulated in many tumours [43]. Interestingly, the generation of L1-70 in the plasma membrane is accompanied with the shedding of a soluble form of approximately 135 kDa (L1-135) into the extracellular space (see [107] and [61], Figure 2). The functions of this fragment are still unknown.

**Figure 3:**
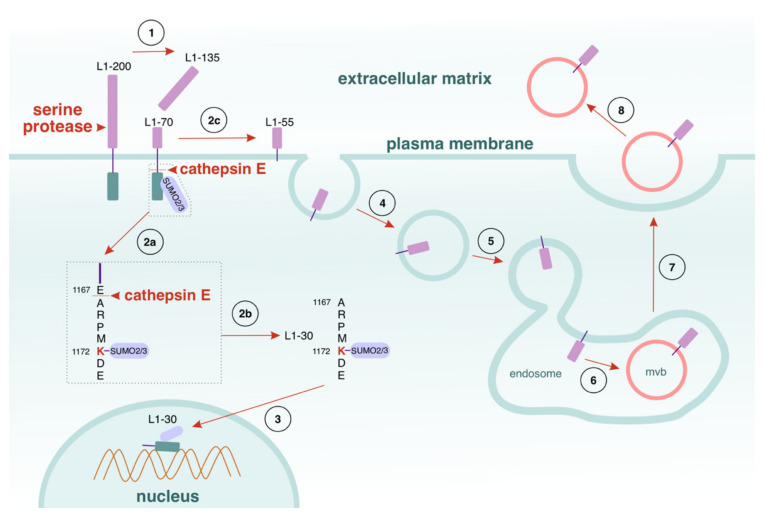
The formation and intracellular pathways of L1-30. (1) After generation from full-length L1 (L1-200), L1-70 is sumoylated by sumo-2 and/or sumo-3, and becomes a substrate for the enzyme cathepsin E, (2a) which cleaves L1 at E_1167_. As a result, two new fragments are generated: a soluble L1-30 fragment (2b) and L1-55 (2c). (3) Once discharged into the cytoplasm, L1-30 enters the nucleus. (4, 5) L1-55 remains bound to the plasmalemma, and from there it is directed to the late endosomes (6). In the endosomes, L1-55 is embedded into multivesicular bodies (mvb) and subjected to exocytosis into the ECM (***7***) by exosomes (8).

Further studies of intracellular L1-fragments have reported that the administration of a function-triggering L1 antibody to dissociated cerebellar neurons initiates the formation of a sumoylated 30 kDa L1 fragment (L1-30) by cathepsin E [110]. L1-30 enters the nucleus [110] (Figure 3).

Modification of the sumoylation site at K_1172_, or the cathepsin E cleavage site at E_1167_, eradicates the formation of L1-30, whereas alteration of the nuclear localisation signal at K_1147_ averts the nuclear internalisation of the fragment, but not its generation. Additionally, L1-30 production can be blocked by pepstatin, an aspartyl protease inhibitor, which also inhibits the L1-induced migration of cerebellar neurons and Schwann cells in dorsal root ganglia, thus impairing axonal myelination [110]. However, the application of L1 agonists has a stimulatory effect on both neural cell types [111,112]. Mutation of the cathepsin E cleavage site of HEK293 cells obstructs their L1-stimulated migration. However, migration is abolished upon silencing of cathepsin E, and enhanced by overexpression of the enzyme [110]. These observations are indicative of the importance of L1-30 for proper cell migration and axonal myelination.

When serine proteases and cathepsin E cleave L1 at the plasma membrane, another membrane-bound fragment of approximately 55 kDa (L1-55) is generated [110]: see Figure 3. L1-55 is directed to the late endosomes, then embedded into multivesicular bodies and released into the ECM by exosomes (Figure 3). The functions of L1-55, similarly to those of L1-135, need further experimental attention.

## 5. Sumoylation of L1 Affects the Generation of Proteolytic Fragments

Interestingly, the post-translational modification of L1 may determine which fragments can be proteolytically generated [68,110]. In a controlled fashion, homophilic interactions trigger the generation of a transmembrane 70 kDa and an intracellular 30 kDa L1 fragment. Notably, sumoylation regulates the direction of proteolysis of L1: only the sumo-2/3-modified 70 kDa fragment could be cleaved by cathepsin E to a 30 kDa portion (Figure 3). Although sumoylation bears a resemblance to ubiquitination, the processes are not identical [113]. Even though sumo-1 and sumo-2/3 activate the same enzymatic conjugation [114], they have different roles, because they interact with diverse target proteins and conjugate them with different isoforms [115,116]. Sumoylation orchestrates a broad spectrum of processes related to the target proteins, both under normal conditions and in response to a variety of pathologies: protein activity, degradation, interactions and localisation, nucleo-cytoplasmic trafficking, DNA repair, and transcription [117,118,119]. Sumoylation is also crucial for the control of neuronal motility and axonal guidance during development, as well as for the normal functioning of the nervous system [120,121,122]. L1-70 and L1-30 follow different intracellular routes: L1-70 enters the nucleus via the endosomes–cytosol path, whereas L1-30 is directed into the cytosol after its generation (Figure 2 and Figure 3). These findings indicate that sumoylation can modify the intracellular destiny of proteolytic fragments; moreover, if sumoylation is abolished, the nuclear import of the fragments is impaired as well. As mentioned above, the 30 kDa L1 fragment is implicated in neuronal migration [110], while the 70 kDa fragment stimulates neuritogenesis and is associated with development, regeneration, and plasticity in the nervous system. Additionally, their occurrence in the nucleus suggests that they have an effector role in nuclear events. As recently shown in vivo, by cleaving L1CAM and producing L1-70, MBP triggers a cascade that suppresses neuron-differentiation-associated gene expression and activates Erk1/2 by PPARγ2 [123]. This novel pathway, described by Yan et al., promotes axonal outgrowth and significantly ameliorates functional recovery from spinal cord injury [123].

## 6. Nuclear Binding Partners of L1

The nuclear presence of L1 fragments implies possible interactions with other molecules. In the search for motifs in L1 that are known to mediate possible interactions between L1 and nuclear molecules, Kraus et al. [124] identified one LXXLL motif (L_1136_LILL) in the transmembrane domain and one FXXLF motif (F_1046_HILF) in the fifth FNIII-like sequence of L1. L_1136_LILL is also present in the co-regulators (co-activators and co-repressors) of nuclear receptors, which are DNA-binding transcription factors essential for the development, differentiation, and metabolism of the eukaryotic cells [125,126,127]. The transcription factors are further categorised as follows: Class I, comprising the steroid receptor family, i.e., receptors for progesterone, estrogens, androgen, glucocorticoid, and mineralocorticoid; Class II, which includes receptors of the thyroid/retinoid group (peroxisome proliferator-activated receptors, receptors for thyroid hormones, vitamins D and A); and Class III, represented by the orphan receptors. There is a significant similarity between the two motifs FXXLF and LXXLL, and they probably contribute to the stabilisation of the ligand–nuclear receptor complex [128,129,130,131,132]. L1-70 contains both the LXXLL and FXXLF motifs; therefore, studies have investigated whether these motifs are involved in the interaction with the nuclear receptors. Indeed, both motifs in the extracellular and transmembrane domain of this L1 fragment facilitate interactions with the nuclear estrogen receptors α and β, peroxisome proliferator-activated receptor γ, and retinoid X receptor β [124]. Alterations in LXXLL and FXXLF disturb the interaction between L1 and the nuclear receptors. Indeed, the introduction of the mutated forms into embryonic mice cerebella in utero resulted in compromised motor coordination and motor learning. Additionally, this impaired synaptic functioning very much resembles the impairment typical of L1-deficient mouse [27]. Therefore, we can conclude that synaptogenesis and synaptic plasticity depend on the interaction between nuclear L1 and distinct nuclear receptors.

Recently, a new potential binding partner of the intracellular L1 domain has been identified (included into the previously mentioned fragment L1-55) [133]. L1-55 binds directly to methyl CpG binding protein 2 (MeCP2) via the sequence motif KDET, and, thus, MeCP2 regulates some L1-dependent processes, including neurite outgrowth and neuronal migration. These combined observations are in agreement with the previously reported nuclear localisation of other receptor molecules. Already in 1993, a proposed cleavage model for Notch was shown to be essential for underlying Notch signalling upon activation by ligands [134,135,136]. After that, several other transmembrane proteins, such as receptor tyrosine kinases [137,138,139] and fibrocystin [140,141], were shown to be proteolytically cleaved, thus giving rise to nuclear fragments, which convey receptor signalling to the nucleus. CD146 (also known as cell surface glycoprotein MUC18 and the melanoma-associated cell adhesion molecule) has been observed in the cytosol and nucleus of endothelial progenitors and neuroblastoma cells [142,143]. Unlike the long CD146 isoform, which is found predominantly in the cytosol, the short CD146 isoform is primarily translocated to the nucleus [142,144]. Such a difference in the localisation of the two CD146 isoforms is indicative of their specific functions; the proliferation and migration of epithelial progenitor cells is promoted by the short CD146 fragment, while the long CD146 fragment leads to the later stabilisation of capillaries [144,145,146].

All these observations raise the question of whether the cleavage and nuclear translocation of fragments can be seen also for other L1 family members. Notably, NCAM is proteolytically cleaved by matrix metalloproteases at the plasma membrane [147,148]. When the function-triggering NCAM antibody and a peptide comprising the effector domain of myristoylated alanine-rich C kinase substrate are administered, their interaction with PSA leads to generation of the subsequent fragment at the plasmalemma and their translocation into the nucleus [149]. Similarly to the fragments of L1, the transmembrane NCAM fragment loaded with PSA moves into the nucleus when neurons are stimulated with surrogate NCAM ligands in vitro [147]. The enhancement of this process is linked to mutations in the clock-related genes, after the PSA deprivation of the dissociated neurons by specific enzymes. There is a circadian oscillation of the nuclear PSA levels in different brain regions, and these changes influence clock-related gene expression, as shown in vivo in the mouse cerebellum and suprachiasmatic nucleus [147]. These studies suggest that not only the protein backbone of the NCAM fragment but also attached carbohydrates co-entering the cell nucleus contribute to specific functions of the carrier protein [106]. Do carbohydrates on L1 also contribute to L1 proteolysis and the functions of the resulting fragments?

## 7. The Proteolysis of L1, and the Application of L1 Mimetics Stimulating Proteolysis, Contribute to the Regeneration of the Injured Nervous System

Another provocative question is whether the third FNIII-like domain of L1, which is a target of many proteases, can be used as a potential therapeutic stimulator of L1-specific functions. To answer this question, Schulz et al. [150] used a 22-mer murine L1 peptide from the third FNIII-like domain for a covalent conjugation to gold nanoparticles (AuNPs). The authors aimed to obtain functionalised particles that trigger homophilically cognate and beneficial L1-mediated functions. The peptide–AuNP conjugate was achieved by combination of two cysteine-terminated forms of FNIII peptide: a derivate of L1, and small thiolated poly(ethylene) glycol (PEG) ligands that reacted with citrate stabilised AuNPs of 14 and 40 nm in diameter. The layer composition of the functionalised AuNPs was optimised by adjusting the proportions of the mixed components for the induction of homophilic interactions. These optimised peptide/PEG–AuNPs were kept stable in artificial cerebrospinal fluid over the course of 30 days, and were able to interact with the extracellular part of L1 on both neuronal and Schwann cells, as observed in L1-deficient and non-mutant mice by means of different cell-based assays. In vitro, the L1-functionalised particles had a stimulating effect on Schwan cells and neurons [150]. These findings raised confidence that AuNPs functionalised with the peptides from the third FNIII-like domain of L1, which is a target of various proteases, have the potential to increase the effectiveness of the other therapeutic strategies for the treatment of nervous system injuries. Nevertheless, further experiments should aim to minimize the biological stimulus size of applicable L1 towards the size of small molecules, which are bound to diffuse better in injured tissue than nanoparticles.

Kataria et al. [111] identified eight small molecule L1 agonists that enhance the proteolysis of L1, and thus, levels of membrane-bound proteolytic and nuclear L1 fragments in cell-based assays and in vivo. These agonists stimulated all the processes mediated by L1 [111]; for instance, severed femoral nerves remyelinated and regenerated rapidly when those molecules were applied. In particular, in a murine model of spinal cord injuries, a restoration of the monoaminergic innervation and suppression of astrogliosis and microglia activity was observed [111]. Such improvements are correlated with the enhanced expression of L1-proteolytic fragments after treatment with L1 agonists, compared with non-treated or mock-treated injured spinal cords [111]. Small organic compounds that bind to L1 and stimulate beneficial homophilic L1 functions seem to increase L1 proteolysis, thus opening another window to novel strategies in the treatment of injured nervous systems.

## 8. Conclusions

Experimental evidence gathered in recent years sheds light on the great functional diversity of L1 and its fragments that emerge in the process of proteolysis. L1 not only mediates adhesion between cells, but can also promote cell motility when cleaved into fragments by several proteases. Now, it is clear that proteolysis is not a sign of degradation; rather, it contributes to the functional heterogeneity of L1, together with distinct post-translational modifications of the cleaved fragments. Carbohydrates attached to the protein backbone and co-entering the cell nucleus contribute to specific functions of the carrier molecule/fragment. Moreover, carbohydrates can affect sumoylation and the intracellular fate of proteolytic fragments, and shape morphogenic events that are required not only for proper development but also for the regeneration of the nervous system. Taken together, these data indicate that targeting the proteolysis of cell adhesion molecules is a promising tool for therapy of the acute or chronically injured nervous system.

## Data Availability

Not applicable.

## References

[1] Kleene R., Schachner M. (2004). Glycans and neural cell interactions. Nat. Rev. Neurosci..

[2] Crossin K.L., Krushel L.A. (2000). Cellular signaling by neural cell adhesion molecules of the immunoglobulin superfamily. Dev. Dyn..

[3] Sytnyk V., Leshchyns’ka I., Schachner M. (2017). Neural Cell Adhesion Molecules of the Immunoglobulin Superfamily Regulate Synapse Formation, Maintenance, and Function. Trends Neurosci..

[4] Cope E.C., Gould E. (2019). Adult Neurogenesis, Glia, and the Extracellular Matrix. Cell Stem Cell.

[5] Südhof T.C. (2021). The cell biology of synapse formation. J. Cell Biol..

[6] Jiang X., Sando R., Südhof T.C. (2021). Multiple signaling pathways are essential for synapse formation induced by synaptic adhesion molecules. Proc. Natl. Acad. Sci. USA.

[7] Brümmendorf T., Kenwrick S., Rathjen F.G. (1998). Neural cell recognition molecule L1: From cell biology to human hereditary brain malformations. Curr. Opin. Neurobiol..

[8] Keilhauer G., Faissner A., Schachner M. (1985). Differential inhibition of neurone-neurone, neurone-astrocyte and astrocyte-astrocyte adhesion by L1, L2 and N-CAM antibodies. Nature.

[9] Maness P.F., Schachner M. (2007). Neural recognition molecules of the immunoglobulin superfamily: Signaling transducers of axon guidance and neuronal migration. Nat. Neurosci..

[10] Nagaraj V., Mikhail M., Baronio M. (2022). Antagonistic L1 Adhesion Molecule Mimetic Compounds Inhibit Glioblastoma Cell Migration In Vitro. Biomolecules.

[11] Law J.W., Lee A.Y., Sun M., Nikonenko A.G., Chung S.K., Dityatev A., Schachner M., Morellini F. (2003). Decreased anxiety, altered place learning, and increased CA1 basal excitatory synaptic transmission in mice with conditional ablation of the neural cell adhesion molecule L1. J. Neurosci..

[12] Conacci-Sorrell M., Kaplan A., Raveh S., Gavert N., Sakurai T., Ben-Ze’ev A. (2005). The shed ectodomain of Nr-CAM stimulates cell proliferation and motility, and confers cell transformation. Cancer Res..

[13] Wang S., Allen R.J., Fang S.Y., Li P. (2017). Cross-modal working memory binding and L1-L2 word learning. Mem. Cogn..

[14] Duncan B.W., Murphy K.E., Maness P.F. (2021). Molecular Mechanisms of L1 and NCAM Adhesion Molecules in Synaptic Pruning, Plasticity, and Stabilization. Front. Cell Dev. Biol..

[15] Vos Y.J., Hofstra R.M. (2010). An updated and upgraded L1CAM mutation database. Hum. Mutat..

[16] Schäfer M.K., Nam Y.C., Moumen A., Keglowich L., Bouché E., Küffner M., Bock H.H., Rathjen F.G., Raoul C., Frotscher M. (2010). L1 syndrome mutations impair neuronal L1 function at different levels by divergent mechanisms. Neurobiol. Dis..

[17] Christaller W.A., Vos Y., Gebre-Medhin S., Hofstra R.M., Schäfer M.K. (2017). L1 syndrome diagnosis complemented with functional analysis of L1CAM variants located to the two N-terminal Ig-like domains. Clin. Genet..

[18] Gauntner T.D., Karumuri M., Guzman M.A., Starnes S.E., Besmer S., Pinz H., Braddock S.R., Andreone T.L. (2021). Hirschsprung Disease in an Infant with L1 syndrome: Report of a New Case and a novel L1CAM variant. Clin. Case Rep..

[19] Dahme M., Bartsch U., Martini R., Anliker B., Schachner M., Mantei N. (1997). Disruption of the mouse L1 gene leads to malformations of the nervous system. Nat. Genet..

[20] Kurumaji A., Nomoto H., Okano T., Toru M. (2001). An association study between polymorphism of L1CAM gene and schizophrenia in a Japanese sample. Am. J. Med. Genet..

[21] Vukojevic V., Mastrandreas P. (2020). Evolutionary conserved role of neural cell adhesion molecule-1 in memory. Transl. Psychiatry.

[22] Loers G., Appel D., Lutz D., Congiu L., Kleene R. (2021). Amelioration of the abnormal phenotype of a new L1 syndrome mouse mutation with L1 mimetics. FASEB J..

[23] Chidsey B.A., Baldwin E.E., Toydemir R., Ahles L., Hanson H., Stevenson D.A. (2014). L1CAM whole gene deletion in a child with L1 syndrome. Am. J. Med. Genetics. Part A.

[24] Cohen N.R., Taylor J.S., Scott L.B., Guillery R.W., Soriano P., Furley A.J. (1998). Errors in corticospinal axon guidance in mice lacking the neural cell adhesion molecule L1. Curr. Biol..

[25] Rünker A.E., Bartsch U., Nave K.A., Schachner M. (2003). The C264Y missense mutation in the extracellular domain of L1 impairs protein trafficking in vitro and in vivo. J. Neurosci..

[26] Ourednik J., Ourednik V., Bastmeyer M., Schachner M. (2001). Ectopic expression of the neural cell adhesion molecule L1 in astrocytes leads to changes in the development of the corticospinal tract. Eur. J. Neurosci..

[27] Fransen E., D’Hooge R., Van Camp G., Verhoye M., Sijbers J., Reyniers E., Soriano P., Kamiguchi H., Willemsen R., Koekkoek S.K. (1998). L1 knockout mice show dilated ventricles, vermis hypoplasia and impaired exploration patterns. Hum. Mol. Genet..

[28] De Angelis E., Watkins A., Schäfer M., Brümmendorf T., Kenwrick S. (2002). Disease-associated mutations in L1 CAM interfere with ligand interactions and cell-surface expression. Hum. Mol. Genet..

[29] Michelson P., Hartwig C., Schachner M., Gal A., Veske A., Finckh U. (2002). Missense mutations in the extracellular domain of the human neural cell adhesion molecule L1 reduce neurite outgrowth of murine cerebellar neurons. Hum. Mutat..

[30] Marx M., Diestel S., Bozon M., Keglowich L., Drouot N., Bouché E., Frebourg T., Minz M., Saugier-Veber P., Castellani V. (2012). Pathomechanistic characterization of two exonic L1CAM variants located in trans in an obligate carrier of X-linked hydrocephalus. Neurogenetics.

[31] Bateman A., Jouet M., MacFarlane J., Du J.S., Kenwrick S., Chothia C. (1996). Outline structure of the human L1 cell adhesion molecule and the sites where mutations cause neurological disorders. EMBO J..

[32] Moos M., Tacke R., Scherer H., Teplow D., Früh K., Schachner M. (1988). Neural adhesion molecule L1 as a member of the immunoglobulin superfamily with binding domains similar to fibronectin. Nature.

[33] Sawaya M.R., Wojtowicz W.M., Andre I., Qian B., Wu W., Baker D., Eisenberg D., Zipursky S.L. (2008). A double S shape provides the structural basis for the extraordinary binding specificity of Dscam isoforms. Cell.

[34] Felding-Habermann B., Silletti S., Mei F., Siu C.H., Yip P.M., Brooks P.C., Cheresh D.A., O’Toole T.E., Ginsberg M.H., Montgomery A.M. (1997). A single immunoglobulin-like domain of the human neural cell adhesion molecule L1 supports adhesion by multiple vascular and platelet integrins. J. Cell Biol..

[35] Schachner M. (1997). Neural recognition molecules and synaptic plasticity. Curr. Opin. Cell Biol..

[36] Schäfer M.K., Altevogt P. (2010). L1CAM malfunction in the nervous system and human carcinomas. Cell. Mol. Life Sci..

[37] Rader C., Kunz B., Lierheimer R., Giger R.J., Berger P., Tittmann P., Gross H., Sonderegger P. (1996). Implications for the domain arrangement of axonin-1 derived from the mapping of its NgCAM binding site. EMBO J..

[38] Kadmon G., Altevogt P. (1997). The cell adhesion molecule L1: Species- and cell-type-dependent multiple binding mechanisms. Differ. Res. Biol. Divers..

[39] Kleene R., Yang H., Kutsche M., Schachner M. (2001). The neural recognition molecule L1 is a sialic acid-binding lectin for CD24, which induces promotion and inhibition of neurite outgrowth. J. Biol. Chem..

[40] Acheson A., Sunshine J.L., Rutishauser U. (1991). NCAM polysialic acid can regulate both cell-cell and cell-substrate interactions. J. Cell Biol..

[41] Wei C.H., Ryu S.E. (2012). Homophilic interaction of the L1 family of cell adhesion molecules. Exp. Mol. Med..

[42] Schäfer M.K., Schmitz B., Diestel S. (2010). L1CAM ubiquitination facilitates its lysosomal degradation. FEBS Lett..

[43] Kiefel H., Bondong S., Hazin J., Ridinger J., Schirmer U., Riedle S., Altevogt P. (2012). L1CAM: A major driver for tumor cell invasion and motility. Cell Adhes. Migr..

[44] Hua T., Liu S., Xin X., Jin Z., Liu Q., Chi S., Wang X., Wang H. (2016). Prognostic significance of L1 cell adhesion molecule in cancer patients: A systematic review and meta-analysis. Oncotarget.

[45] Guo M., Gong H., Nie D., Li Z. (2021). High L1CAM expression predicts poor prognosis of patients with endometrial cancer: A systematic review and meta-analysis. Medicine.

[46] Chu L.Y., Guo D.M., Chen J.T., Fang W.K., Xie J.J., Peng Y.H., Xu Y.W., Li X.X. (2020). The Diagnostic Value of Serum L1CAM in Patients With Colorectal Cancer. Technol. Cancer Res. Treat..

[47] Zander H., Rawnaq T., von Wedemeyer M., Tachezy M., Kunkel M., Wolters G., Bockhorn M., Schachner M., Izbicki J.R., Kaifi J. (2011). Circulating levels of cell adhesion molecule L1 as a prognostic marker in gastrointestinal stromal tumor patients. BMC Cancer.

[48] Ganesh K., Basnet H., Kaygusuz Y., Laughney A.M., He L., Sharma R., O’Rourke K.P., Reuter V.P., Huang Y.H., Turkekul M. (2020). L1CAM defines the regenerative origin of metastasis-initiating cells in colorectal cancer. Nat. Cancer.

[49] Friedl P., Mayor R. (2017). Tuning Collective Cell Migration by Cell-Cell Junction Regulation. Cold Spring Harb. Perspect. Biol..

[50] Angiolini F., Cavallaro U. (2017). The Pleiotropic Role of L1CAM in Tumor Vasculature. Int. J. Mol. Sci..

[51] Angiolini F., Belloni E., Giordano M., Campioni M., Forneris F., Paronetto M.P., Lupia M., Brandas C., Pradella D., Di Matteo A. (2019). A novel L1CAM isoform with angiogenic activity generated by NOVA2-mediated alternative splicing. eLife.

[52] Fogel M., Gutwein P., Mechtersheimer S., Riedle S., Stoeck A., Smirnov A., Edler L., Ben-Arie A., Huszar M., Altevogt P. (2003). L1 expression as a predictor of progression and survival in patients with uterine and ovarian carcinomas. Lancet.

[53] Maretzky T., Schulte M., Ludwig A., Rose-John S., Blobel C., Hartmann D., Altevogt P., Saftig P., Reiss K. (2005). L1 is sequentially processed by two differently activated metalloproteases and presenilin/gamma-secretase and regulates neural cell adhesion, cell migration, and neurite outgrowth. Mol. Cell. Biol..

[54] Er E.E., Valiente M., Ganesh K., Zou Y., Agrawal S., Hu J., Griscom B., Rosenblum M., Boire A., Brogi E. (2018). Pericyte-like spreading by disseminated cancer cells activates YAP and MRTF for metastatic colonization. Nat. Cell Biol..

[55] Strekalova H., Buhmann C., Kleene R., Eggers C., Saffell J., Hemperly J., Weiller C., Müller-Thomsen T., Schachner M. (2006). Elevated levels of neural recognition molecule L1 in the cerebrospinal fluid of patients with Alzheimer disease and other dementia syndromes. Neurobiol. Aging.

[56] Beer S., Oleszewski M., Gutwein P., Geiger C., Altevogt P. (1999). Metalloproteinase-mediated release of the ectodomain of L1 adhesion molecule. J. Cell Sci..

[57] Kuhn P.H., Koroniak K., Hogl S., Colombo A., Zeitschel U., Willem M., Volbracht C., Schepers U., Imhof A., Hoffmeister A. (2012). Secretome protein enrichment identifies physiological BACE1 protease substrates in neurons. EMBO J..

[58] Zhou L., Barão S., Laga M., Bockstael K., Borgers M., Gijsen H., Annaert W., Moechars D., Mercken M., Gevaert K. (2012). The neural cell adhesion molecules L1 and CHL1 are cleaved by BACE1 protease in vivo. J. Biol. Chem..

[59] Linneberg C., Toft C.L.F., Kjaer-Sorensen K., Laursen L.S. (2019). L1cam-mediated developmental processes of the nervous system are differentially regulated by proteolytic processing. Sci. Rep..

[60] Nayeem N., Silletti S., Yang X., Lemmon V.P., Reisfeld R.A., Stallcup W.B., Montgomery A.M. (1999). A potential role for the plasmin(ogen) system in the posttranslational cleavage of the neural cell adhesion molecule L1. J. Cell Sci..

[61] Kleene R., Lutz D., Loers G., Bork U., Borgmeyer U., Hermans-Borgmeyer I., Schachner M. (2021). Revisiting the proteolytic processing of cell adhesion molecule L1. J. Neurochem..

[62] Mechtersheimer S., Gutwein P., Agmon-Levin N., Stoeck A., Oleszewski M., Riedle S., Postina R., Fahrenholz F., Fogel M., Lemmon V. (2001). Ectodomain shedding of L1 adhesion molecule promotes cell migration by autocrine binding to integrins. J. Cell Biol..

[63] Riedle S., Kiefel H., Gast D., Bondong S., Wolterink S., Gutwein P., Altevogt P. (2009). Nuclear translocation and signalling of L1-CAM in human carcinoma cells requires ADAM10 and presenilin/gamma-secretase activity. Biochem. J..

[64] Gutwein P., Oleszewski M., Mechtersheimer S., Agmon-Levin N., Krauss K., Altevogt P. (2000). Role of Src kinases in the ADAM-mediated release of L1 adhesion molecule from human tumor cells. J. Biol. Chem..

[65] Hennen E., Safina D., Haussmann U., Wörsdörfer P., Edenhofer F., Poetsch A., Faissner A. (2013). A LewisX glycoprotein screen identifies the low density lipoprotein receptor-related protein 1 (LRP1) as a modulator of oligodendrogenesis in mice. J. Biol. Chem..

[66] Lieberoth A., Splittstoesser F., Katagihallimath N., Jakovcevski I., Loers G., Ranscht B., Karagogeos D., Schachner M., Kleene R. (2009). Lewis(x) and alpha2,3-sialyl glycans and their receptors TAG-1, Contactin, and L1 mediate CD24-dependent neurite outgrowth. J. Neurosci..

[67] Lutz D., Kataria H., Kleene R., Loers G., Chaudhary H., Guseva D., Wu B., Jakovcevski I., Schachner M. (2016). Myelin Basic Protein Cleaves Cell Adhesion Molecule L1 and Improves Regeneration After Injury. Mol. Neurobiol..

[68] Lutz D., Loers G., Kleene R., Oezen I., Kataria H., Katagihallimath N., Braren I., Harauz G., Schachner M. (2014). Myelin basic protein cleaves cell adhesion molecule L1 and promotes neuritogenesis and cell survival. J. Biol. Chem..

[69] Readhead C., Hood L. (1990). The dysmyelinating mouse mutations shiverer (shi) and myelin deficient (shimld). Behav. Genet..

[70] Berger T., Rubner P., Schautzer F., Egg R., Ulmer H., Mayringer I., Dilitz E., Deisenhammer F., Reindl M. (2003). Antimyelin antibodies as a predictor of clinically definite multiple sclerosis after a first demyelinating event. N. Engl. J. Med..

[71] Boggs J.M. (2006). Myelin basic protein: A multifunctional protein. Cell. Mol. Life Sci..

[72] Krugmann B., Radulescu A., Appavou M.S., Koutsioubas A., Stingaciu L.R., Dulle M., Förster S., Stadler A.M. (2020). Membrane stiffness and myelin basic protein binding strength as molecular origin of multiple sclerosis. Sci. Rep..

[73] Martinsen V., Kursula P. (2022). Multiple sclerosis and myelin basic protein: Insights into protein disorder and disease. Amino Acids.

[74] Wolf M.K., Billings-Gagliardi S. (1984). CNS hypomyelinated mutant mice (jimpy, shiverer, quaking): In vitro evidence for primary oligodendrocyte defects. Adv. Exp. Med. Biol..

[75] Landry C.F., Ellison J.A., Pribyl T.M., Campagnoni C., Kampf K., Campagnoni A.T. (1996). Myelin basic protein gene expression in neurons: Developmental and regional changes in protein targeting within neuronal nuclei, cell bodies, and processes. J. Neurosci..

[76] Roonprapunt C., Huang W., Grill R., Friedlander D., Grumet M., Chen S., Schachner M., Young W. (2003). Soluble cell adhesion molecule L1-Fc promotes locomotor recovery in rats after spinal cord injury. J. Neurotrauma.

[77] Lutz D., Sharaf A., Drexler D., Kataria H. (2017). Proteolytic cleavage of transmembrane cell adhesion molecule L1 by extracellular matrix molecule Reelin is important for mouse brain development. Sci. Rep..

[78] Howell B.W., Hawkes R., Soriano P., Cooper J.A. (1997). Neuronal position in the developing brain is regulated by mouse disabled-1. Nature.

[79] Sheldon M., Rice D.S., D’Arcangelo G., Yoneshima H., Nakajima K., Mikoshiba K., Howell B.W., Cooper J.A., Goldowitz D., Curran T. (1997). Scrambler and yotari disrupt the disabled gene and produce a reeler-like phenotype in mice. Nature.

[80] D’Arcangelo G., Homayouni R., Keshvara L., Rice D.S., Sheldon M., Curran T. (1999). Reelin is a ligand for lipoprotein receptors. Neuron.

[81] Howell B.W., Herrick T.M., Cooper J.A. (1999). Reelin-induced tyrosine [corrected] phosphorylation of disabled 1 during neuronal positioning. Genes Dev..

[82] Trommsdorff M., Gotthardt M., Hiesberger T., Shelton J., Stockinger W., Nimpf J., Hammer R.E., Richardson J.A., Herz J. (1999). Reeler/Disabled-like disruption of neuronal migration in knockout mice lacking the VLDL receptor and ApoE receptor 2. Cell.

[83] Franco S.J., Martinez-Garay I., Gil-Sanz C., Harkins-Perry S.R., Müller U. (2011). Reelin regulates cadherin function via Dab1/Rap1 to control neuronal migration and lamination in the neocortex. Neuron.

[84] Quattrocchi C.C., Wannenes F., Persico A.M., Ciafré S.A., D’Arcangelo G., Farace M.G., Keller F. (2002). Reelin is a serine protease of the extracellular matrix. J. Biol. Chem..

[85] Devanathan V., Jakovcevski I., Santuccione A., Li S., Lee H.J., Peles E., Leshchyns’ka I., Sytnyk V., Schachner M. (2010). Cellular form of prion protein inhibits Reelin-mediated shedding of Caspr from the neuronal cell surface to potentiate Caspr-mediated inhibition of neurite outgrowth. J. Neurosci..

[86] Jossin Y. (2020). Reelin Functions, Mechanisms of Action and Signaling Pathways During Brain Development and Maturation. Biomolecules.

[87] Lambert de Rouvroit C., de Bergeyck V., Cortvrindt C., Bar I., Eeckhout Y., Goffinet A.M. (1999). Reelin, the extracellular matrix protein deficient in reeler mutant mice, is processed by a metalloproteinase. Exp. Neurol..

[88] Lugli G., Krueger J.M., Davis J.M., Persico A.M., Keller F., Smalheiser N.R. (2003). Methodological factors influencing measurement and processing of plasma reelin in humans. BMC Biochem..

[89] Jossin Y., Gui L., Goffinet A.M. (2007). Processing of Reelin by embryonic neurons is important for function in tissue but not in dissociated cultured neurons. J. Neurosci..

[90] Hisanaga A., Morishita S., Suzuki K., Sasaki K., Koie M., Kohno T., Hattori M. (2012). A disintegrin and metalloproteinase with thrombospondin motifs 4 (ADAMTS-4) cleaves Reelin in an isoform-dependent manner. FEBS Lett..

[91] Krstic D., Rodriguez M., Knuesel I. (2012). Regulated proteolytic processing of Reelin through interplay of tissue plasminogen activator (tPA), ADAMTS-4, ADAMTS-5, and their modulators. PLoS ONE.

[92] Ignatova N., Sindic C.J., Goffinet A.M. (2004). Characterization of the various forms of the Reelin protein in the cerebrospinal fluid of normal subjects and in neurological diseases. Neurobiol. Dis..

[93] Jossin Y., Ignatova N., Hiesberger T., Herz J., Lambert de Rouvroit C., Goffinet A.M. (2004). The central fragment of Reelin, generated by proteolytic processing in vivo, is critical to its function during cortical plate development. J. Neurosci..

[94] Yamakage Y., Kato M., Hongo A., Ogino H., Ishii K., Ishizuka T., Kamei T., Tsuiji H., Miyamoto T., Oishi H. (2019). A disintegrin and metalloproteinase with thrombospondin motifs 2 cleaves and inactivates Reelin in the postnatal cerebral cortex and hippocampus, but not in the cerebellum. Mol. Cell. Neurosci..

[95] Turk L.S., Kuang X., Dal Pozzo V., Patel K., Chen M., Huynh K., Currie M.J., Mitchell D., Dobson R.C.J., D’Arcangelo G. (2021). The structure-function relationship of a signaling-competent, dimeric Reelin fragment. Structure.

[96] Mata-Balaguer T., Cuchillo-Ibañez I., Calero M., Ferrer I., Sáez-Valero J. (2018). Decreased generation of C-terminal fragments of ApoER2 and increased reelin expression in Alzheimer’s disease. FASEB J..

[97] Ogino H., Yamakage Y., Yamashita M.B., Kohno T., Hattori M. (2020). Assay for Reelin-Cleaving Activity of ADAMTS and Detection of Reelin and Its Fragments in the Brain. Methods Mol. Biol..

[98] Nagae M., Suzuki K., Yasui N. (2021). Structural studies of reelin N-terminal region provides insights into a unique structural arrangement and functional multimerization. J. Biochem..

[99] Zhao S., Frotscher M. (2010). Go or stop? Divergent roles of Reelin in radial neuronal migration. Neuroscientist.

[100] Endo A., Hashimoto K., Takada Y., Takada A. (1999). The activation of the tissue plasminogen activator-plasmin system induced in the mouse hippocampus after injection of trimethyltin: Possible proteolysis of highly polysialylated NCAM. Jpn. J. Physiol..

[101] Bajor M., Kaczmarek L. (2013). Proteolytic remodeling of the synaptic cell adhesion molecules (CAMs) by metzincins in synaptic plasticity. Neurochem. Res..

[102] Seidenfaden R., Krauter A., Schertzinger F., Gerardy-Schahn R., Hildebrandt H. (2003). Polysialic acid directs tumor cell growth by controlling heterophilic neural cell adhesion molecule interactions. Mol. Cell. Biol..

[103] Amoureux M.C., Coulibaly B., Chinot O., Loundou A., Metellus P., Rougon G., Figarella-Branger D. (2010). Polysialic acid neural cell adhesion molecule (PSA-NCAM) is an adverse prognosis factor in glioblastoma, and regulates olig2 expression in glioma cell lines. BMC Cancer.

[104] Glass J.D., Watanabe M., Fedorkova L., Shen H., Ungers G., Rutishauser U. (2003). Dynamic regulation of polysialylated neural cell adhesion molecule in the suprachiasmatic nucleus. Neuroscience.

[105] Shen H., Watanabe M., Tomasiewicz H., Glass J.D. (2001). Genetic deletions of NCAM and PSA impair circadian function in the mouse. Physiol. Behav..

[106] Westphal N., Theis T. (2017). Nuclear fragments of the neural cell adhesion molecule NCAM with or without polysialic acid differentially regulate gene expression. Sci. Rep..

[107] Lutz D., Wolters-Eisfeld G., Joshi G., Djogo N., Jakovcevski I., Schachner M., Kleene R. (2012). Generation and nuclear translocation of sumoylated transmembrane fragment of cell adhesion molecule L1. J. Biol. Chem..

[108] Zhang Y., Yeh J., Richardson P.M., Bo X. (2008). Cell adhesion molecules of the immunoglobulin superfamily in axonal regeneration and neural repair. Restor. Neurol. Neurosci..

[109] Hu J., Lin S.L., Schachner M. (2022). A fragment of cell adhesion molecule L1 reduces amyloid-β plaques in a mouse model of Alzheimer’s disease. Cell Death Dis..

[110] Lutz D., Wolters-Eisfeld G., Schachner M., Kleene R. (2014). Cathepsin E generates a sumoylated intracellular fragment of the cell adhesion molecule L1 to promote neuronal and Schwann cell migration as well as myelination. J. Neurochem..

[111] Kataria H., Lutz D., Chaudhary H., Schachner M., Loers G. (2016). Small Molecule Agonists of Cell Adhesion Molecule L1 Mimic L1 Functions In Vivo. Mol. Neurobiol..

[112] Li R., Sahu S., Schachner M. (2018). Phenelzine, a cell adhesion molecule L1 mimetic small organic compound, promotes functional recovery and axonal regrowth in spinal cord-injured zebrafish. Pharmacol. Biochem. Behav..

[113] Geiss-Friedlander R., Melchior F. (2007). Concepts in sumoylation: A decade on. Nat. Rev. Mol. Cell Biol..

[114] Tatham M.H., Jaffray E., Vaughan O.A., Desterro J.M., Botting C.H., Naismith J.H., Hay R.T. (2001). Polymeric chains of SUMO-2 and SUMO-3 are conjugated to protein substrates by SAE1/SAE2 and Ubc9. J. Biol. Chem..

[115] Vertegaal A.C., Andersen J.S., Ogg S.C., Hay R.T., Mann M., Lamond A.I. (2006). Distinct and overlapping sets of SUMO-1 and SUMO-2 target proteins revealed by quantitative proteomics. Mol. Cell. Proteom..

[116] Saitoh H., Hinchey J. (2000). Functional heterogeneity of small ubiquitin-related protein modifiers SUMO-1 versus SUMO-2/3. J. Biol. Chem..

[117] Peters M., Wielsch B., Boltze J. (2017). The role of SUMOylation in cerebral hypoxia and ischemia. Neurochem. Int..

[118] Chang H.M., Yeh E.T.H. (2020). SUMO: From Bench to Bedside. Physiol. Rev..

[119] Filippopoulou C., Simos G., Chachami G. (2020). The Role of Sumoylation in the Response to Hypoxia: An Overview. Cells.

[120] Kadaré G., Toutant M., Formstecher E., Corvol J.C., Carnaud M., Boutterin M.C., Girault J.A. (2003). PIAS1-mediated sumoylation of focal adhesion kinase activates its autophosphorylation. J. Biol. Chem..

[121] Martin S., Nishimune A., Mellor J.R., Henley J.M. (2007). SUMOylation regulates kainate-receptor-mediated synaptic transmission. Nature.

[122] Ripamonti S., Shomroni O. (2020). SUMOylation controls the neurodevelopmental function of the transcription factor Zbtb20. J. Neurochem..

[123] Yan Z., Chu L., Jia X., Lin L., Cheng S. (2021). Myelin basic protein enhances axonal regeneration from neural progenitor cells. Cell Biosci..

[124] Kraus K., Kleene R., Henis M., Braren I., Kataria H., Sharaf A., Loers G., Schachner M., Lutz D. (2018). A Fragment of Adhesion Molecule L1 Binds to Nuclear Receptors to Regulate Synaptic Plasticity and Motor Coordination. Mol. Neurobiol..

[125] Smirnov A.N. (2002). Nuclear receptors: Nomenclature, ligands, mechanisms of their effects on gene expression. Biochemistry.

[126] Gronemeyer H., Gustafsson J.A., Laudet V. (2004). Principles for modulation of the nuclear receptor superfamily. Nat. Rev. Drug Discov..

[127] Bain D.L., Heneghan A.F., Connaghan-Jones K.D., Miura M.T. (2007). Nuclear receptor structure: Implications for function. Annu. Rev. Physiol..

[128] He B., Kemppainen J.A., Wilson E.M. (2000). FXXLF and WXXLF sequences mediate the NH2-terminal interaction with the ligand binding domain of the androgen receptor. J. Biol. Chem..

[129] He B., Bowen N.T., Minges J.T., Wilson E.M. (2001). Androgen-induced NH2- and COOH-terminal Interaction Inhibits p160 coactivator recruitment by activation function 2. J. Biol. Chem..

[130] He B., Minges J.T., Lee L.W., Wilson E.M. (2002). The FXXLF motif mediates androgen receptor-specific interactions with coregulators. J. Biol. Chem..

[131] Dubbink H.J., Hersmus R., Pike A.C., Molier M., Brinkmann A.O., Jenster G., Trapman J. (2006). Androgen receptor ligand-binding domain interaction and nuclear receptor specificity of FXXLF and LXXLL motifs as determined by L/F swapping. Mol. Endocrinol..

[132] Askew E.B., Minges J.T., Hnat A.T., Wilson E.M. (2012). Structural features discriminate androgen receptor N/C terminal and coactivator interactions. Mol. Cell. Endocrinol..

[133] Loers G., Kleene R., Girbes Minguez M. (2022). The Cell Adhesion Molecule L1 Interacts with Methyl CpG Binding Protein 2 via Its Intracellular Domain. Int. J. Mol. Sci..

[134] Lieber T., Kidd S., Alcamo E., Corbin V., Young M.W. (1993). Antineurogenic phenotypes induced by truncated Notch proteins indicate a role in signal transduction and may point to a novel function for Notch in nuclei. Genes Dev..

[135] Struhl G., Fitzgerald K., Greenwald I. (1993). Intrinsic activity of the Lin-12 and Notch intracellular domains in vivo. Cell.

[136] Struhl G., Greenwald I. (2001). Presenilin-mediated transmembrane cleavage is required for Notch signal transduction in Drosophila. Proc. Natl. Acad. Sci. USA.

[137] Yarden Y., Sliwkowski M.X. (2001). Untangling the ErbB signalling network. Nat. Rev. Mol. Cell Biol..

[138] Wang S.C., Hung M.C. (2009). Nuclear translocation of the epidermal growth factor receptor family membrane tyrosine kinase receptors. Clin. Cancer Res..

[139] Chen M.K., Hung M.C. (2015). Proteolytic cleavage, trafficking, and functions of nuclear receptor tyrosine kinases. FEBS J..

[140] Hiesberger T., Gourley E., Erickson A., Koulen P., Ward C.J., Masyuk T.V., Larusso N.F., Harris P.C., Igarashi P. (2006). Proteolytic cleavage and nuclear translocation of fibrocystin is regulated by intracellular Ca^2+^ and activation of protein kinase C. J. Biol. Chem..

[141] Outeda P., Menezes L., Hartung E.A., Bridges S., Zhou F., Zhu X., Xu H., Huang Q., Yao Q., Qian F. (2017). A novel model of autosomal recessive polycystic kidney questions the role of the fibrocystin C-terminus in disease mechanism. Kidney Int..

[142] Kebir A., Harhouri K., Guillet B., Liu J.W., Foucault-Bertaud A., Lamy E., Kaspi E., Elganfoud N., Vely F., Sabatier F. (2010). CD146 short isoform increases the proangiogenic potential of endothelial progenitor cells in vitro and in vivo. Circ. Res..

[143] Obu S., Umeda K. (2021). CD146 is a potential immunotarget for neuroblastoma. Cancer Sci..

[144] Harhouri K., Kebir A., Guillet B., Foucault-Bertaud A., Voytenko S., Piercecchi-Marti M.D., Berenguer C., Lamy E., Vely F., Pisano P. (2010). Soluble CD146 displays angiogenic properties and promotes neovascularization in experimental hind-limb ischemia. Blood.

[145] Stalin J., Harhouri K., Hubert L., Garrigue P., Nollet M., Essaadi A., Muller A., Foucault-Bertaud A., Bachelier R., Sabatier F. (2016). Soluble CD146 boosts therapeutic effect of endothelial progenitors through proteolytic processing of short CD146 isoform. Cardiovasc. Res..

[146] Nollet M., Bachelier R., Joshkon A., Traboulsi W., Mahieux A., Moyon A., Muller A., Somasundaram I., Simoncini S., Peiretti F. (2022). Involvement of Multiple Variants of Soluble CD146 in Systemic Sclerosis: Identification of a Novel Profibrotic Factor. Arthritis Rheumatol..

[147] Westphal N., Kleene R., Lutz D., Theis T., Schachner M. (2016). Polysialic acid enters the cell nucleus attached to a fragment of the neural cell adhesion molecule NCAM to regulate the circadian rhythm in mouse brain. Mol. Cell. Neurosci..

[148] Westphal N., Loers G., Lutz D., Theis T. (2017). Generation and intracellular trafficking of a polysialic acid-carrying fragment of the neural cell adhesion molecule NCAM to the cell nucleus. Sci Rep..

[149] Theis T., Mishra B., von der Ohe M., Loers G., Prondzynski M., Pless O., Blackshear P.J., Schachner M., Kleene R. (2013). Functional role of the interaction between polysialic acid and myristoylated alanine-rich C kinase substrate at the plasma membrane. J. Biol. Chem..

[150] Schulz F., Lutz D., Rusche N., Bastús N.G., Stieben M., Höltig M., Grüner F., Weller H., Schachner M., Vossmeyer T. (2013). Gold nanoparticles functionalized with a fragment of the neural cell adhesion molecule L1 stimulate L1-mediated functions. Nanoscale.

